# A Primer on the Clinical Aspects of Sarcoidosis for the Basic and Translational Scientist

**DOI:** 10.3390/jcm10132857

**Published:** 2021-06-28

**Authors:** Marc A. Judson

**Affiliations:** Division of Pulmonary and Critical Care Medicine, Albany Medical College, Albany, NY 12208, USA; judsonm@amc.edu

**Keywords:** sarcoidosis, immunopathogenesis, phenotype, clinical manifestations, risk factors, treatment

## Abstract

The immunopathogenesis of sarcoidosis remains unclear. This failure in understanding has been clinically impactful, as it has impeded the accurate diagnosis, treatment, and prevention of this disease. Unraveling the mechanisms of sarcoidosis will require input from basic and translational scientists. In order to reach this goal, scientists must have a firm grasp of the clinical aspects of the disease, including its diagnostic criteria, the immunologic defects, clinical presentations, response to therapy, risk factors, and clinical course. This manuscript will provide an overview of the clinical aspects of sarcoidosis that are particularly relevant for the basic and translational scientist. The variable phenotypic expression of the disease will be described, which may be integral in identifying immunologic disease mechanisms that may be relevant to subgroups of sarcoidosis patients. Data concerning treatment and risk factors may yield important insights concerning germane immunologic pathways involved in the development of disease. It is hoped that this manuscript will stimulate communication between scientists and clinicians that will eventually lead to improved care of sarcoidosis patients.

## 1. Introduction

The clinician at the patient’s bedside and the medical scientific researcher in his laboratory often seem lightyears apart. The medical researcher’s world is one of quantification, objectivity, and reproducibility. The clinician’s world is inexact and, although based in science, is overlaid with complex psychosocial and financial issues. Despite this expansive distance in terms of focus, it is imperative that the medical researcher and clinician communicate in order to achieve medical breakthroughs and improve patient care. Understanding the immunopathogenesis of a disease provides the clinician with the rationale for specific therapy and improves his understanding of ineffective therapy and medication side effects. Understanding the clinical aspects of a disease assists the medical researcher in focusing on mechanisms that are clinically relevant. This manuscript will provide an overview of the clinical aspects of sarcoidosis, focusing on issues that are particularly relevant for the basic and translational scientist. Currently, the immunopathogenesis of sarcoidosis is poorly understood, and treatment of this disease is suboptimal. It is hoped that this clinical review will aid medical researchers in their efforts to understand the immune mechanisms of sarcoidosis that are clinically impactful. Furthermore, we believe that the concepts expressed here could be extrapolated to other granulomatous and immunologic diseases.

## 2. How Understanding the Clinical Aspects of Sarcoidosis Can Assist the Scientist

Understanding the clinical aspects of a disease can aid the scientist in focusing on mechanisms that are consistent with the clinical findings, relevant, and impactful. In the case of sarcoidosis, three such clinical aspects are its phenotypic expression, risk factors, and the effects of drug therapy. Each of these is discussed briefly below in general terms and is described specifically in terms of sarcoidosis in the remainder of the manuscript.

**Figure 1:**
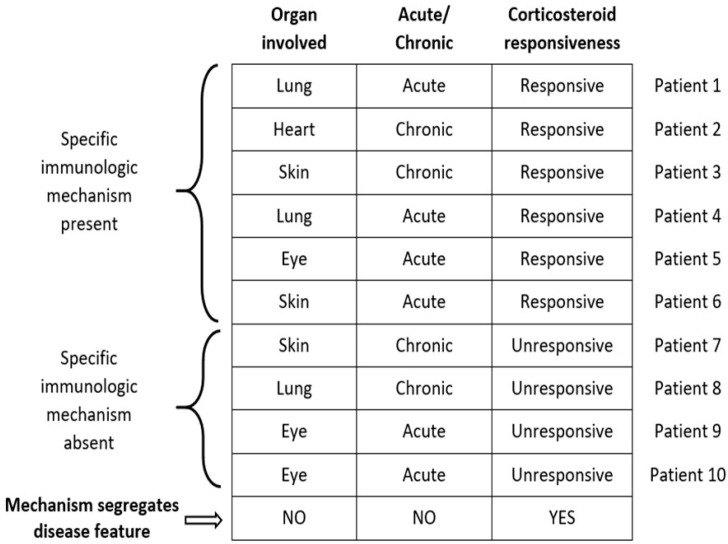
Relationship of immunologic mechanism to sarcoidosis phenotype. An immunologic mechanism may be associated with a particular phenotypic characteristic of sarcoidosis but not others. In the example displayed in the figure, the mechanism distinguishes corticosteroid-responsive from corticosteroid-unresponsive cases. If the cases were only described in terms of sarcoidosis organ involvement or the duration of disease, it would be falsely assumed that the mechanism was not clinically relevant in sarcoidosis. This example justifies the importance of defining clinical phenotypes in detail to fully understand the impact of a purported mechanism of disease. Reproduced with permission from Judson, M.A. Human Immunol 2019; 80:85–89 Reference [1].

*The importance of clinical phenotyping:* The diagnosis of a disease is usually based on the presence of specific historical information, symptoms, laboratory tests, and occasionally, the response to therapy. Although these criteria may be adequate to render a clinical diagnosis, they may correlate poorly with the underlying mechanisms of the disease. A clinical disease may represent a common endpoint for several disparate disease mechanisms. Therefore, a specific mechanism may be responsible for only a fraction of a clinical disease. Clinical phenotyping of a disease may partition patients into cohorts such that a disease mechanism that fails to reveal the cause of a disease may explain the cause of a specific disease phenotype. Figure 1 displays this concept in a theoretical example involving sarcoidosis. In this example, a potential immunologic mechanism of the disease is not associated with all cases of sarcoidosis nor with two phenotypic characteristics (specific organ involvement with sarcoidosis, whether the disease is acute or chronic). However, the mechanism is associated with the phenotypic characteristic of corticosteroid refractory disease. This example emphasizes the importance of using multiple phenotyping techniques to uncover disease mechanisms. 

*Disease risk factors:* The risk of contracting a disease may be increased or decreased because of numerous clinical factors, including age, race, ethnicity, occupational or environmental exposure, diet, and lifestyle. Knowledge of risk and protective factors for a disease may generate clues concerning previously unconsidered pathways. In addition, evidence supporting a proposed disease mechanism would be enhanced if risk factors were shown to stimulate that mechanism and protective factors were shown to blunt it.

*The effect of drug therapy:* Effective drug therapy may provide important clues concerning disease mechanisms by demonstrating that blocking or stimulating various inflammatory pathways lessens disease activity. In addition, drug therapy and other maneuvers that worsen disease activity may uncover important disease mechanisms. It needs to be emphasized that even when an immunologic pathway involved in a disease has been carefully elucidated, its clinical relevance is unclear without performing a drug trial. The immune system is highly redundant, and pathways that are uniformly associated with a disease may not be causal. This explains why sarcoidosis drug trials that target putative immunopathogenic sarcoidosis pathways [2] have sometimes been negative [3]. 

## 3. Common Sarcoidosis Phenotypes

*Organ involvement:* Sarcoidosis is a multisystem granulomatous disease of an unknown cause that can affect every organ. The lung is the most common organ involved with sarcoidosis at a frequency of typically over 90 percent of cases [4,5]. The next most common organs involved with sarcoidosis are the skin, eyes, peripheral lymph nodes, and liver, with rates between 10 and 25 percent in each of these organs [4,5]. The frequency of organ involvement in a large sarcoidosis cohort (*n* = 1248) is shown in Table 1. In this cohort, the mean number of organs involved per patient was 2.33 [4].

It should be noted that the frequency of specific organ involvement is greatly dependent on the method of detection. Rigorous methods of detection may yield much higher frequencies of organ involvement. For example, signs or symptoms of liver sarcoidosis are found in 5–15% of sarcoidosis patients [6,7], liver function test abnormalities are found in up to 35% of sarcoidosis patients [8], and liver biopsies demonstrate granulomatous inflammation in 50–80% of sarcoidosis patients [9]. Usually, rigorous methods to detect all sarcoidosis organ involvements are not used because there are minimal implications of asymptomatic organ involvement, except in the case of eye and cardiac involvement. A corollary concept is that sarcoidosis organ involvement that causes no symptoms probably may remain completely undetected. Indeed, as sarcoidosis often causes no symptoms, the presence of the disease may completely escape detection. This concept is supported by the fact that mass radiographic screening studies have identified relatively high rates of pulmonary sarcoidosis compared to standard epidemiologic surveys [10,11], presumedly by detecting a large number of asymptomatic cases.

The diagnosis of sarcoidosis is based on a compatible clinical presentation, usually (but not always) a tissue biopsy confirming granulomatous inflammation, and the exclusion of alternative causes of granulomatous disease [12]. Common granulomatous diseases confused with sarcoidosis are listed in Table 2. As the criteria for a compatible clinical presentation and the method to exclude alternative diseases are left to the clinician’s discretion, the diagnosis of sarcoidosis is not standardized [12], and is based on arbitrary, subjective criteria [13]. There is some controversy concerning the number of organs that are required to establish the diagnosis of sarcoidosis [14]. As sarcoidosis is considered a multisystem disease, some clinicians have required evidence of granulomatous inflammation in at least two organs for the diagnosis of sarcoidosis to be secured [15]. However, it is not unusual for the diagnosis of sarcoidosis to be established on the basis of granulomatous inflammation of just one organ in the proper clinical context [5,16]. Sarcoidosis is undoubtedly a systemic disease by virtue of its association with anergy, polyclonal gammopathy, specific inflammatory syndromes (e.g., erythema nodosum) [17], and the recurrence of sarcoidosis in allografts of organ transplant recipients with sarcoidosis [18]. However, these systemic features of disease could be present in a patient with only one organ involved with granulomatous inflammation. Of course, because sarcoidosis organ involvement often causes no symptoms and thereby escapes detection, many of these patients assumed to have just one organ involved with the disease may have additional organs involved. 

Non-pulmonary sarcoidosis has been estimated to occur in approximately 8 percent of sarcoidosis patients [19]. Nearly half of non-pulmonary sarcoidosis patients have skin involvement, and half of these have isolated skin involvement [19]. The skin is second only to the lung in terms of the frequency of isolated sarcoidosis organ involvement. 

### 3.1. Implications for the Scientist

As the lung is the overwhelmingly most common organ to be affected by sarcoidosis and numerous infectious and non-infectious pulmonary granulomatous diseases result from antigens inhaled into the lung, this suggests that pulmonary sarcoidosis develops from an inhaled antigen. The lung may be the “portal of entry” of a putative sarcoidosis antigen that then may “disseminate” into other organs of the body. It is interesting that the skin is second to the lung as a site of isolated organ sarcoidosis. The skin is a particularly conductive site of antigen capture [20] and adaptive immune responses [21]. Therefore, the skin may be a conducive portal of entry for antigens that induce sarcoidosis. 

Sarcoidosis is thought to develop similar to other granulomatous diseases of known cause, where an antigen is detected and processed by antigen detecting cells such as macrophages and dendritic cells [22]. These processed antigens are subsequently presented by human leukocyte antigen (HLA) Class II molecules to a restricted group of T-cell receptors on naïve T lymphocytes that are predominately of the CD4+ class [23]. The interplay of antigen, T-cell receptors, and HLA molecules occurs at the HLA molecule bonding site and is thought to be integral to the development of sarcoidosis [24]. These events are thought to polarize T lymphocytes to a Th1/Th17 phenotype, leading to cellular recruitment, proliferation, and differentiation, leading to the formation of the sarcoid granuloma [25]. However, it is problematic to reconcile this granulomatous mechanism with the dissemination of sarcoidosis granulomas to multiple organs. If sarcoidosis results from a granulomatous response to a foreign antigen, that antigen must disseminate to additional organs to cause disease there. The antigen may disseminate throughout the body through the lymphatic or vascular system. Antigens from infectious pathogens and inorganic substances have been suspected as potential inducers of sarcoidosis. However, as will be discussed, a disseminated infectious organism or inorganic antigen has never been clearly established in sarcoidosis. It is possible that the development of sarcoidosis beyond the portal of entry may result from dysregulation of the immune system leading to autoimmunity rather than the dissemination of an antigen. Autoimmunity in sarcoidosis may occur via molecular mimicry whereby the putative antigens trigger inflammation, leading to exposure of self-peptides [26]. Autoantigen reactivity has been demonstrated in bronchoalveolar lavage and serum from sarcoidosis patients [27]. Specifically, vimentin has been implicated as a T-cell autoantigen in pulmonary sarcoidosis, particularly in the context of HLA-DRB1*03, the Vα2.3/Vβ22 T-cell receptor (TCR), and Löfgren’s syndrome [28,29]. It is also possible that the antigen that leads to the development of sarcoidosis is not responsible for granulomatous inflammation at all, but rather leads to the exposure of self-peptides, causing autoimmunity that is responsible for both granulomas in the initial organ and all subsequent organs. In this sense, the putative antigen of sarcoidosis may function as an adjuvant that non-specifically stimulates the immune system of susceptible individuals to expose self-peptides, leading to the granulomatous inflammation of sarcoidosis. A major unresolved issue underlying this discussion is whether sarcoidosis represents a normal immune response to an exposure or is the result of a deranged immune response. It is also uncertain if autoimmunity is either primarily or secondarily involved in the immunopathogenesis of sarcoidosis. 

As the diagnosis of sarcoidosis is not standardized, there is a reasonable chance that a patient diagnosed with sarcoidosis actually has an alternative granulomatous disease [30]. This should be kept in mind when analyzing clinical or immunologic data from sarcoidosis cohorts. Similarly, because sarcoidosis involvement of various organs is frequently undetected, correlations between specific sarcoidosis organ involvement and immunologic findings are suspect. 

In addition, any robust model of sarcoidosis should account not only for multisystem granulomatous involvement but also the aforementioned non-granulomatous evidence of systemic disease. For example, anergy associated with sarcoidosis needs to be explained. Sarcoidosis has been described as demonstrating an “immune paradox” where there is peripheral anergy while exaggerated inflammation develops at sites of disease [31]. Proposed mechanisms for sarcoidosis anergy include disequilibrium between effector and regulatory T lymphocytes [31], depletion of peripheral blood lymphocytes as lymphocytes traffic to sites of inflammation [32], immunosuppressive effects of CD8+ T cells that accumulate peripherally in sarcoidosis patients with chronic disease [33], and CD4+ T cell exhaustion [34]. The hypergammaglobulinemia seen with sarcoidosis suggests that B cells may be integrally involved in the formation of the sarcoidosis granuloma [35].

**Figure 2:**
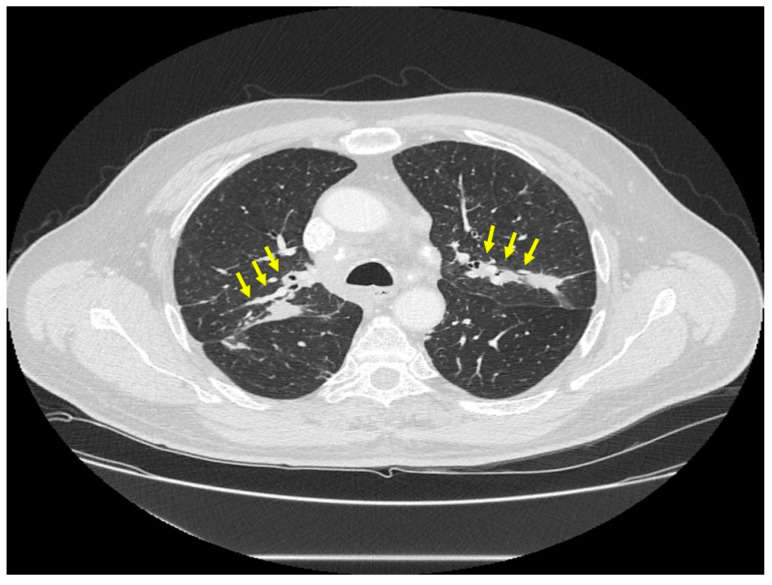
Chest CT scan image of a patient with pulmonary sarcoidosis, demonstrating lung opacities from granulomatous inflammation around airways (arrows).

**Figure 3:**
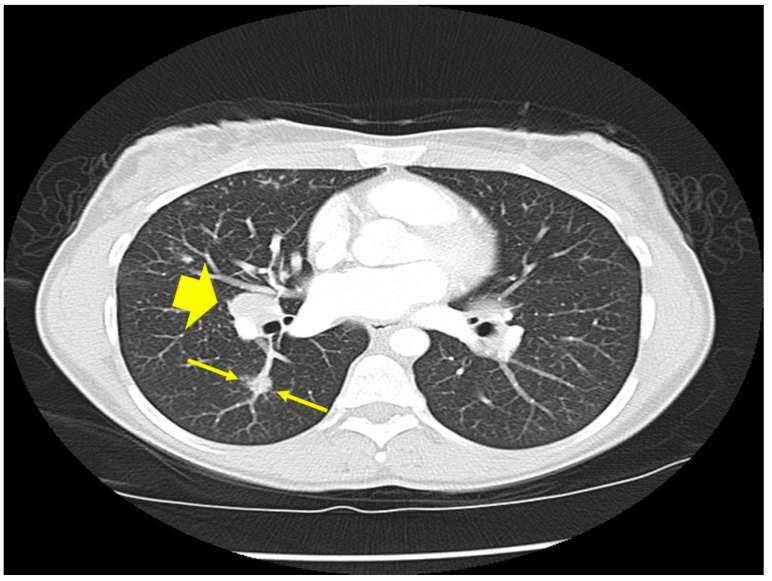
Chest CT scan image of a patient with pulmonary sarcoidosis demonstrating lung opacities from granulomatous inflammation around pulmonary vessels (thin arrows). Scatter parenchymal nodules and hilar adenopathy are seen, especially on the right (thick arrow).

**Figure 4:**
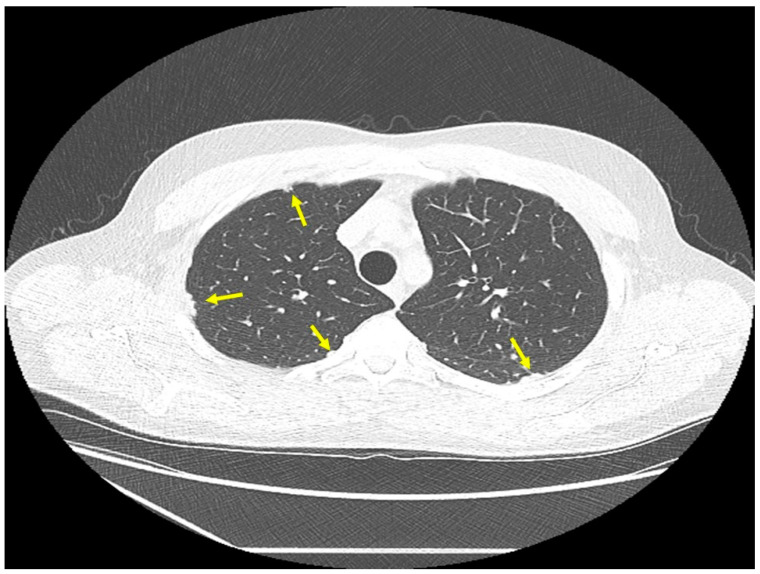
Chest CT image of a patient with pulmonary sarcoidosis, demonstrating subpleural opacities from granulomatous inflammation (arrows).

**Figure 5:**
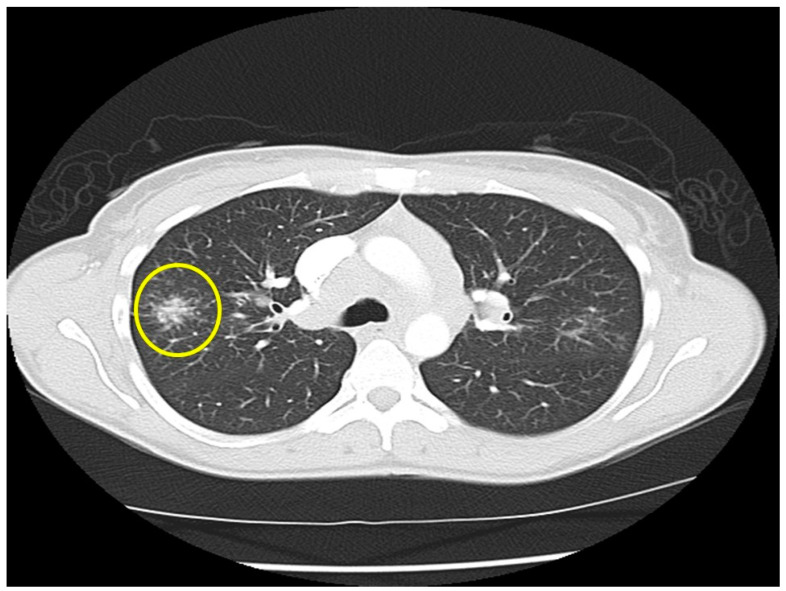
Chest CT image of a “galaxy sign” in a patient with pulmonary sarcoidosis. Micronodules coalescence into a central mass-like lesion (within circle).

**Figure 6:**
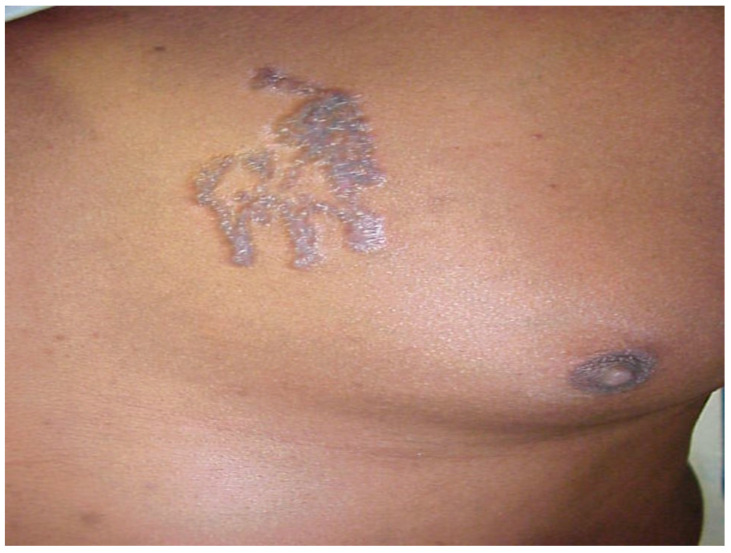
Tattoo of a sarcoidosis patient that was completely replaced by granulomatous inflammation.

**Figure 7:**
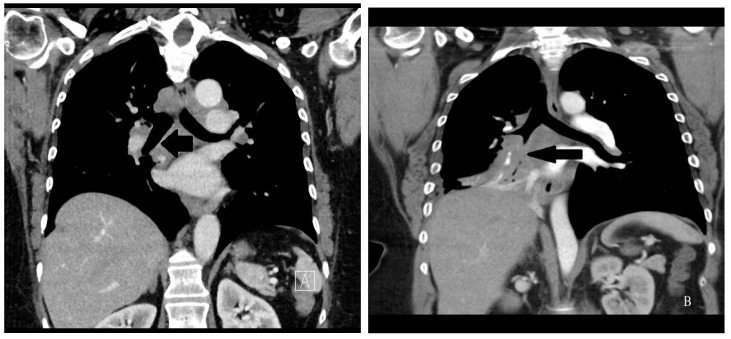
(**A**) Left figure: Chest CT sagittal image of mediastinal lymph nodes from sarcoidosis. The lymph nodes do not appreciably disturb airway patency, and the right bronchus intermedius is completely unobstructed (arrow); (**B**) Right figure: Chest CT sagittal image of mediastinal lymph nodes from small cell lung carcinoma. The lymph nodes do not respect anatomic boundaries and compromise the right bronchus intermedius (arrow).

*Location of sarcoidosis within each organ:* Sarcoidosis has not only a predilection to involve certain organs but to involve certain portions of each organ. Sarcoidosis more commonly involves the upper portions of the lung [36]. In the lung, sarcoid granulomas most commonly form in perilymphatic locations [37,38,39], including around the bronchovascular bundles [40]. Therefore, lesions are common along airways (Figure 2), pulmonary vessels (Figure 3), and in subpleural locations (Figure 4). Sarcoidosis granulomas not only have a predilection for depositing in the pulmonary lymphatic system but also in peripheral and visceral lymph nodes throughout the body as well as the spleen [41,42]. Another highly specific radiographic feature of pulmonary sarcoidosis is the galaxy sign [43], where small micronodules that are apparent in the periphery become more condensed and conglomerate centrally, not unlike the appearance of a globular cluster galaxy (Figure 5). The mediastinal lymph nodes are commonly involved in pulmonary sarcoidosis, with rates often greater than 90 percent [44,45]. Eye sarcoidosis very commonly develops in the uveal tract [46,47], which is the vascular supply of the eye. Perivascular eye involvement is quite notable with sarcoidosis-induced posterior uveitis, which typically manifests as a retinal periphlebitis [46]. This condition results in extensive sheathing and infiltrates exuding from the retinal veins to cause a “candle wax drippings” appearance [48]. Skin sarcoidosis may have a varied appearance and may affect any portion of the skin. However, skin sarcoidosis often develops in scars and tattoos (Figure 6). Sarcoidosis involvement is often most prominent in pericellular locations in tissues. For example, in neurosarcoidosis, a relatively rare form of the disease that may be life-threatening [49], granulomas usually develop as a leptomeningeal involvement that extend along the Virchow–Robin spaces to the epineurium and perineurium [50]. Therefore, neurosarcoidosis granulomas are primarily situated around nerve fascicles and axons rather than within them. These findings are consistent with the longstanding tenet that sarcoid granulomas respect anatomic barriers and vital structures. Another example of this is the chest radiographic appearance of hilar mediastinal lymph nodes from sarcoidosis as “potato nodes”, which owe their appearance to the fact that these nodes do not compress adjacent airways (Figure 7A) as malignant lymph nodes might (Figure 7B). In summary, although sarcoid granulomas may involve any portion of an organ, they have propensity to form around vessels, the lymphatic system, and scarred tissue.

### 3.2. Implications for the Scientist

The predilection for the upper portions of the lung to be involved with pulmonary sarcoidosis suggests that the antigen(s) that is associated with sarcoidosis is inhaled, similar to what is observed with silica and coal workers pneumoconiosis [51]. There is a tendency for sarcoid granulomas to form around blood vessels and lymphatic channels. The granulomatous inflammation of sarcoidosis seems to be concentrated in perivascular and peri-lymphatic locations and causes relatively little intracellular damage. It is possible that sarcoidosis granuloma formation requires exposure to a vascular or lymphatic antigen or possibly a stimulus transported through the lymphatic or vascular system. The fact that sarcoidosis granulomas form in skin scars and tattoos suggest that fibrotic tissue may be another stimulant for sarcoidosis granuloma growth.

*Acute versus chronic disease:* The clinical course of sarcoidosis is highly variable. Patients may present with an acute form of the disease that has a rapid onset and typically resolves in a few months to 1 year [52]. The prototypical acute form of sarcoidosis is Lofgren’s syndrome, which consists of a combination of erythema nodosum skin lesions, bilateral hilar adenopathy on a chest radiograph, fever, and ankle arthritis/periarthritis [53,54]. Lofgren’s syndrome is typically a self-limiting form of sarcoidosis that resolves with or without therapy in months [53,55]. Lofgren’s syndrome is particularly common in Scandinavian sarcoidosis patients [56]. Other forms of sarcoidosis are more chronic and often have a more insidious onset, and may require therapy for more than 1 year [52,57,58]. In a study of more than 800 Finnish and Japanese sarcoidosis patients, 40–80 percent had complete resolution of chest radiographic abnormalities by 5 years after diagnosis [59]. The number of patients who achieved radiographic resolution slowly increased over time. These data suggest that a large percentage of sarcoidosis patients have a chronic disease that may resolve over 5 years in some of them but persist in others. An analysis of 500 hundred sarcoidosis patients from 10 centers across the world found that more than 70 percent of them still required therapy 2 and 5 years after the diagnosis [57].

### 3.3. Implications for the Scientist

The granulomatous inflammation of sarcoidosis may be short-lived or chronic. The mechanisms responsible for the lifespan of the sarcoidosis granuloma have not been clearly explained. If the granulomas of sarcoidosis represent a response to a foreign antigen, a chronic granulomatous response may simply be the result of the persistence of the antigen that cannot be adequately cleared. Serum amyloid A (SAA) has been postulated to be involved in the maintenance of the sarcoidosis granuloma by promoting antigen persistence. SAA has been frequently found within the sarcoid granuloma and is rarely observed with other granulomatous diseases [60,61]. SAA may block antigen eradication in sarcoidosis through protein aggregation and trapping of antigen within a poorly soluble protein matrix [62]. SAA has been shown to enhance the persistence of experimental granulomas in mice [62]. Sarcoidosis disease chronicity is probably much more complex than can be explained by the presence of SAA. Levels of certain inflammatory biomarkers, various gene haplotypes, and T-cell subsets have been associated with the presence and prognosis of various forms of acute and chronic sarcoidosis [52,54,63]. The aforementioned differences in the resolution of sarcoidosis in a Japanese versus Swedish cohort suggest that there are genetic determinants to the resolution of sarcoid granulomas.

*Fibrotic sarcoidosis:* In a minority of pulmonary sarcoidosis cases, perhaps up to 20 percent [4,64], significant fibrosis will develop. This fibrosis will not resolve spontaneously or with anti-granulomatous therapy. It is this fibrosis that usually determines the prognosis of pulmonary sarcoidosis [65], as well as most of the severe complications [66]. As a large percentage of the severe consequences and death from sarcoidosis relate to fibrotic forms of the disease [67,68], there is an unmet need to develop effective anti-fibrotic therapy for sarcoidosis as well as identify biomarkers to predict the development of this complication.

**Figure 8:**
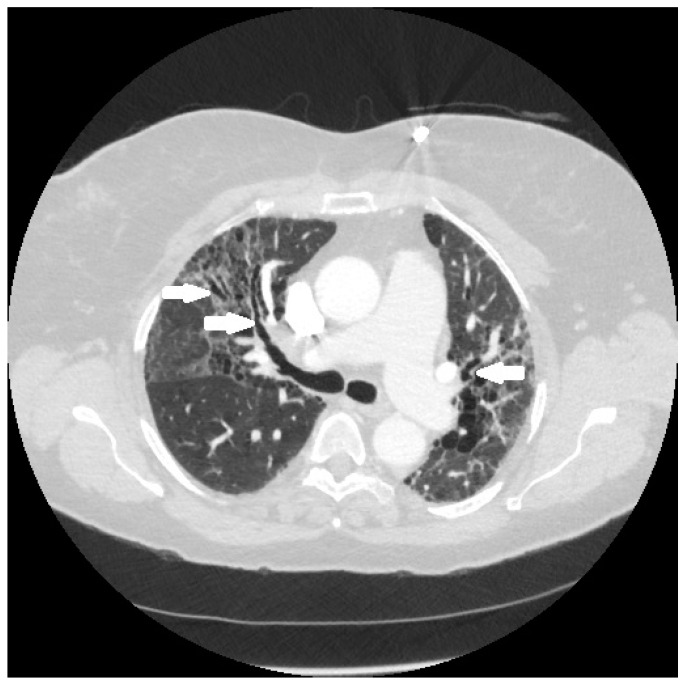
Chest CT scan of fibrotic pulmonary sarcoidosis. The fibrosis is typically in a peribronchial location, which in this case has resulted in fibrosis-induced traction bronchiectasis of airways (arrows).

It is conjectured that the fibrosis in sarcoidosis is the result of granulomatous inflammation [68]. This conjecture is based on three lines of evidence. First, histological examination demonstrates that the majority of the fibrosis develops within or around the granuloma in so-called “hyalinized granulomas [69]. Second, pathological [69] and radiographic analyses [70] have shown that the fibrosis occurs predominantly in peribronchiolar locations, where sarcoid granulomas tend to form (Figure 8). Finally, the fibrosis often coexists with active pulmonary granulomatous inflammation on nuclear imaging studies [71].

### 3.4. Implications for the Scientist

Fibrosis seems to stimulate the growth of sarcoidosis granulomas by their common occurrence in scars and tattoos. It is thought that the initial triggers for many interstitial lung diseases involve a repetitive vascular injury that leads to an aberrant reparatory process, activation of and infiltration of myofibroblasts, and excess deposition of collagen fibrils [72]. Therefore, it is possible that the sarcoid granulomas initially damage the vasculature leading to collagen production and subsequent fibrosis. Such fibrosis may then trigger further granulomatous inflammation. 

Various single nucleotide polymorphisms (SNPs) are associated with fibrotic pulmonary sarcoidosis; these include SNPS for: (a) GREM 1, which encodes for gremlin, a secreted glycoprotein and member of the bone morphogenic proteins [73]; (b) Card15 (caspase recruitment domain-containing protein 15, also known as NOD2 [74], and (c) isoform 3 of transforming growth factor beta (TGF-β3) [75]. Interestingly, the SNP rs35705950, which is strongly associated with idiopathic pulmonary fibrosis [76,77], was not associated with fibrotic forms of sarcoidosis [78]. This suggests that the fibrotic mechanisms identified in other lung diseases may not be reliably extrapolated to sarcoidosis.

## 4. Sarcoidosis Risk Factors

*Demographics:* Sarcoidosis occurs all over the world. However, it is more common in certain ethnic groups, races, and locations. The annual incidence of sarcoidosis is more common in African Americans than Caucasian Americans by a ratio of between 2:1 to 3:1 [79,80]. In addition, the age onset of the disease is approximately 10 years younger in African Americans than Caucasian Americans [4]. African Americans also have more severe disease than Caucasian Americans by virtue of a greater number of organs involved [4], a greater need of anti-sarcoidosis medications [4], a worse Scadding radiographic stage [4], and a greater density of granulomas on tissue biopsy [81]. In the United States, the incidence and prevalence rates of sarcoidosis in Hispanic whites are roughly half of non-Hispanic whites, and the rates in Asian Americans are lower than in Hispanic whites [80]. The highest incidence and prevalence rates of sarcoidosis occur in African Americans and in Nordic countries with incidence rates of 10–20/100,000, and prevalence rates of 140–160/100,000 [80,82]. Higher rates of sarcoidosis have been observed in Northern latitudes such as Northern Europe and Northern Japan [82,83]. In addition, certain phenotypic expressions of sarcoidosis are more common in certain ethnic and racial groups, such as cardiac sarcoidosis in the Japanese [84] and Lofgren’s syndrome in Northern Europeans [85].

Although the incidence and prevalence rates of sarcoidosis are uniformly found to be 50–100% higher in women than men across all racial groups in the United States [80], the gender differences have been reported to be significantly less in non-US locations where men account for 37–50% of cases [82,86,87,88,89]. There are phenotypic differences between the sexes, including that men tend to be diagnosed at an earlier age than women [82], and there are differences in the frequency of certain organ involvement [4].

Although the literature is rife with statements that age of onset of sarcoidosis peaks between 20 and 45 years [15], most recent epidemiologic analyses using large numbers of incident cases have shown that the bulk of new sarcoidosis cases occur after age 45 [80,82]. Sarcoidosis is extremely rare to develop before 18 years of age, with an incidence rate of 0.6–1/100,000 [90].

Several epidemiologic studies have demonstrated that first degree relatives of sarcoidosis patients have an increased risk of having sarcoidosis with odds ratios of 2.5 to 3.8 [91]. A recent analysis of more than 23,000 Swedish sarcoidosis patients found that having at least one first degree relative with sarcoidosis was associated with a 3.7-fold risk of sarcoidosis, and the heritability was 39%.

### 4.1. Implications for the Scientist

These data concerning racial differences and a propensity for sarcoidosis to aggregate in families suggest that genetic factors are important in the development of sarcoidosis. It is hypothesized that sarcoidosis results from exposure to an environmental antigen in a genetically susceptible individual [5,92]. As mentioned, sarcoidosis may be similar to other granulomatous diseases where HLA Class II molecules present processed antigens to T-lymphocyte receptors. Various HLA polymorphisms have been associated with the development of sarcoidosis, protection from sarcoidosis, and various phenotypes of sarcoidosis [93,94]. Furthermore, genome-wide association studies have identified numerous non-HLA genes that are associated with sarcoidosis. These include NOTCH4, which regulates the activity of T cell immune responses [95]; annexin, which affects cell division and apoptosis [96]; and BTNL2, which is involved in T cell activation [95,97].

### 4.2. Environmental Risk Factors

Sarcoidosis is associated with exposure to numerous environmental risk factors. As the exact immunopathogenesis of sarcoidosis is unknown, it is not clear whether these exposures are truly causing sarcoidosis, rendering the immune system more susceptible to developing sarcoidosis, exacerbating subclinical cases of sarcoidosis, or causing a granulomatous disease that is distinct from sarcoidosis [22]. Table 3 lists various exposures that have been associated with the development of sarcoidosis. Common exposures include metal dusts, combustible wood products, and other combustible products, such as World Trade Center disaster dust, organic dusts, and living in a farm environment. Several space-time analyses have shown that sarcoidosis is statistically more likely to develop in the spring than in other seasons of the year. In a study of more than 700 sarcoidosis patients that examined sarcoidosis phenotypes based on environmental exposure, it was shown that certain exposures, including agricultural dusts and wood burning, were associated with a greater likelihood of developing isolated pulmonary sarcoidosis [98]. This suggests that there may be differences between systemic versus pulmonary-only sarcoidosis in terms of exposures. In an analysis of more than 7,000,000 death certificates that also had Occupational Classification Codes recorded where nearly 4000 of them were from sarcoidosis patients, sarcoidosis patients who were exposed to putative etiologic agents had a significantly increased mortality odds ratio than non-sarcoidosis patients without similar exposures [99]. There were significant differences in exposure-associated mortality by sex and race. These data suggest that there may be an interaction between exposure, demographics, and mortality in sarcoidosis.

Infectious agents have also been implicated as a potential cause of sarcoidosis, although the data supporting this conjecture are inconsistent and unconvincing. There is an abundance of indirect evidence that mycobacteria are associated with sarcoidosis. Two meta-analyses of studies evaluating potential infectious diseases that may cause sarcoidosis suggested an etiologic link between mycobacteria and sarcoidosis [123,124]. Molecular techniques have identified mycobacterial components in sarcoidosis tissues in some [125,126,127] but not all [128,129] analyses. In particular, mycobacteria catalase-peroxidase protein (mKatG) has been implicated as associated with sarcoidosis in numerous reports. T-cell responses to mKatG have been observed in peripheral blood monocytes [130,131] and in T-cells in bronchoalveolar lavage fluid of sarcoidosis patients [131,132]. There is evidence that Propionibacterium acnes, a skin commensal bacterium, is associated with sarcoidosis. Several studies have identified specific immune responses to this organism with little to no responses in control subjects [132,133]. Propionibacterium acnes is the only organism that has been cultured from sarcoidosis lesions [133,134]. It is problematic to comprehend how sarcoidosis could be caused by an infectious pathogen, as several immunosuppressive agents are effective sarcoidosis treatments. It is much more plausible that an antigen of an infectious agent might stimulate the immune system to cause the granulomatous inflammation of sarcoidosis.

Cigarette smoking has been shown to be protective of sarcoidosis in several sarcoidosis cohorts [114,135]. A study of non-nicotine cigarette smoking suggests that nicotine is not the constituent that is protective of sarcoidosis [136].

### 4.3. Implications for the Scientist

The exposure data provides strong evidence that an environmental antigen is an integral factor in the development of the sarcoid granuloma. As most of these exposures are airborne, it is likely that most of these environmental antigens enter the lung initially and lead to a granulomatous lung disease. The fact that numerous environmental exposures are associated with sarcoidosis suggests that either the disease results from the downstream result of many possible causes or that sarcoidosis actually represents a myriad of similar diseases, each with its own specific trigger (“the sarcoidoses” [137]). It is likely that genetics plays a role in determining which antigen will trigger a granulomatous sarcoid response. Various HLA polymorphisms may be important in this regard as antigens may only be able to bind to specific ones. Analyses have shown that HLA polymorphisms associated with sarcoidosis are associated with significant changes in the binding pockets of the HLA molecules [94]. It may be that individual antigens require specific HLA molecules to present them to T-lymphocytes to induce the granulomatous process. Evidence supporting this proposed sarcoidosis mechanism relates to chronic beryllium disease (CBD), a granulomatous disorder that is radiographically and histologically indistinguishable from sarcoidosis [138]. CBD is associated with a glutamic acid substitution at position 69 of the HLA-DBP1 chain and position 71 of the HLA-DRB-1 chain [139,140]. Beryllium-specific oligoclonal CD4+ T lymphocytes then recognize beryllium in an MHC Class II-restricted manner (usually via E69 or E71), leading to CD4+ lymphocyte proliferation, recruitment of other T cells and monocytes into the lung, and the production of Th1 cytokines that eventually form granulomas [141]. This mechanism is strikingly similar to that proposed for sarcoidosis. 

The different rates and phenotypic expressions of sarcoidosis between the sexes may relate to hormonal or other immunologic differences. However, these differences may also be the result of differing rates of exposure to associated antigens. In a large sarcoidosis mortality study, men who died of sarcoidosis were more likely to have sarcoidosis-related inhalation exposures, whereas women who died of sarcoidosis were more likely to have been in occupations associated with sarcoidosis that had great person-to-person contact (administration, banking) [99]. These findings are consistent with the fact that women have more skin sarcoidosis than men [142], whereas men have more isolated pulmonary exposure than women [98].

## 5. Drug Treatment of Sarcoidosis and Drug Inducers of Sarcoidosis: Sarcoidosis Pharmacotherapy

The decision to treat sarcoidosis is complex because the disease may be self-limited, cause no symptoms or functional impairment, and treatment is associated with significant toxicity. The indications for treatment have been distilled down to two: (a) significant quality of life impairment and (b) significant risk of a dangerous outcome [143]. Corticosteroids are considered the drug of choice for the granulomatous inflammation of sarcoidosis as they are highly effective and work relatively quickly [144], usually in the order of weeks [145,146]. However, because of a substantial risk of corticosteroid toxicity, alternative agents are commonly used for the treatment of sarcoidosis as corticosteroid-sparing or corticosteroid-replacing therapy [147,148]. Drugs that have shown efficacy for sarcoidosis other than corticosteroids have included (a) antimetabolites-methotrexate [149], azathioprine [150], leflunomide [151]; (b) antimalarials chloroquine [152] and hydroxychloroquine [152]; and (c) tumor necrosis alpha (TNF-α) antagonists [153,154].

### Implications for the Scientist

The effectiveness of corticosteroids in sarcoidosis reveals little concerning the inflammatory pathways involved in sarcoidosis, as corticosteroids suppress numerous inflammatory genes that are activated in chronic inflammatory states [155]. The effectiveness of corticosteroids and other immunosuppressive drugs in sarcoidosis suggests that sarcoidosis is not a typical pathogenic infection that should tend to worsen from such medications. This does not preclude sarcoidosis being the result of a granulomatous response to antigens of infectious agents. The effectiveness of antimetabolites that impede DNA synthesis also does not greatly assist in identifying a specific mechanism involved with sarcoidosis, as these drugs impede DNA synthesis, which should inhibit granuloma formation and cell proliferation from a variety of causes.

The fact that TNF-α antagonists have been useful for the treatment of sarcoidosis is consistent with the belief that this mediator is important in the development of the sarcoidosis granuloma [156]. TNF-α is secreted from macrophages of sarcoidosis patients [157], and sarcoidosis patients with corticosteroid-refractory disease tend to have high concentrations of TNF-α in bronchoalveolar lavage fluid [158]. Furthermore, a meta-analysis has demonstrated a significant association of the −308 TNF-α polymorphism with sarcoidosis compared to controls [159], and other studies also found that the −307 haplotype is associated with good prognosis and a −857 T haplotype associated with persistent disease [160].

## 6. Drug-Induced Sarcoidosis-Like Reactions (DISRs)

A drug-induced sarcoidosis-like reaction (DISR) is defined as a systemic granulomatous reaction that is indistinguishable from sarcoidosis that occurs in a temporal relationship with the initiation of an offending drug [161]. The clinical presentation and histological findings of sarcoidosis and a DISR are identical [161,162]. Similar to the previous discussion concerning exposures associated with sarcoidosis, it is unclear if DISR results from a drug causing sarcoidosis, rendering the immune system more susceptible to developing sarcoidosis, exacerbating a subclinical case of sarcoidosis, or causing a condition that is distinct from sarcoidosis [161]. Common drugs that are associated with DISRs include highly active antiretroviral therapy (HAART), immune checkpoint inhibitors (ICIs), TNF-α antagonists, and interferons (IFNs) [163].

An autoimmune inflammatory syndrome induced by adjuvants (ASIA) is another type of sarcoidosis-like reaction [164,165]. ASIAs are thought to occur by adjuvants stimulating immune pathways and preventing antigens from being degraded, thereby prolonging antigen exposure to antigen-presenting cells [165]. Adjuvants associate with ASIAs include silicone [166], mineral oil [167], and hyaluronic acid [168]. ASIAs have been reported after silicone breast augmentation [169,170] and have resolved after removal of the implant [171].

### Implications for the Scientist

DISRs may yield important insights into the mechanisms responsible for developing sarcoidosis. ICIs block the immune checkpoint pathway site, including neutralizing the cytotoxic T lymphocyte antigen-4 (CTLA-4), programmed cell death protein 1 (PD-1), and the programmed death ligand 1 (PD-L1). ICIs not only enhance anti-tumor activity but also stimulate the immune system resulting in immune-related adverse events (irAEs) [172]. irAEs include rheumatoid arthritis, Sjorgen syndrome, psoriatic arthritis, immune thrombocytopenia, seronegative polyarthritis, and DISRs [173,174]. ICIs that block CTLA-4 can inhibit cluster of differentiation (CD) 80 and CD 86 on antigen-presenting cells that may block T-cell signaling, prolong T-cell activation, and restore T-cell proliferation. The resulting T-cell proliferation and increased expression of T-helper-1 (Th1) markers [175] could induce a DISR because Th1 cells are abundant in sarcoidosis granulomas and are thought to be integral to their development [176]. ICIs that block CTLA-4 have also been shown to increase the number and function of Th17 cells [177] that are also thought to be essential in the formation of the sarcoidosis granuloma [178]. It would seem paradoxical that PD-1 inhibitor ICIs could cause a DISR, as the PD-1 pathway was found to be upregulated in active sarcoidosis, and downregulation of PD-1 expression on CD4+ T cells was associated with spontaneous remission of sarcoidosis [179]. However, similar to CLTA-4 inhibitors, PD-1 inhibitors can increase the number and function of Th17 cells [180,181] and possibly cause a DISR on that basis. 

Highly active retroviral therapy (HAART) induced DISRs are immune reconstitution inflammatory syndrome reactions that occur in patients with HIV infection [182]. HAART results in an increased number of CD4+ T-cells that may lead to the induction of a granulomatous response to specific antigens resulting in a DISR. INF is thought to be important in the development of sarcoidosis by causing Th-1 polarization and Th2 inactivation with increased production of granuloma-promoting cytokines [23,183]. TNF-α plays a significant role in the formation and maintenance of the sarcoidosis granuloma [184], and it is therefore not surprising that TNF-α antagonist drugs are effective anti-sarcoidosis agents. Therefore, it might seem paradoxical that TNF-α antagonists are common drugs associated with DISRs [153,154]. Postulated mechanisms to explain TNF-α induced DISRs include (a) antibodies to soluble TNF-α or soluble TNF-α receptor that could permit the activation of specific autoreactive T-cells; and (b) an imbalance in cytokine production including unopposed IFN production promoting a shift toward a Th1/Th2 profile [185].

## 7. Summary

Sarcoidosis is a disease that is not rigorously defined. It may represent the common pathway of many diseases or, alternatively, represent a collection of distinct diseases. The disease appears to involve a causative exposure in a genetically susceptible individual, and various combinations of exposures and genetic profiles may result in varying phenotypic expression. This makes the elucidation of immunopathogenic mechanisms of sarcoidosis problematic. Scientific exploration of sarcoidosis will require rigorous attention to the clinical aspects of the disease, potential environmental exposures, genetics, and immunology. Given this situation, it seems essential that a collaboration between the clinician and the scientist will be essential in unraveling the cause of sarcoidosis. The clinician’s contribution to this process includes defining clinical phenotypes, identifying risk factors and protective factors, and exploring pharmacologic and other maneuvers that improve or worsen the disease. It is hoped that multidisciplinary collaboration will result in impactful insights that will improve the quality of life of sarcoidosis patients.

## Figures and Tables

**Table 1:** Frequency of sarcoidosis organ involvement in a large cohort *.

Organ	White (*n* = 429)	Black (*n* = 819)	Total (*n* = 1248)
	*n*, (%)	*n*, (%)	*n*, (%)
Lung	363 (84)	749 (91)	1112 (89)
Neurologic	28 (7)	85 (10)	113 (9)
Peripheral lymph node	52 (12)	102 (13)	154 (12)
Kidney	3 (1)	8 (1)	11 (1)
Heart	17 (4)	39 (5)	56 (4)
Skin	97 (23)	305 (37)	402 (32)
Eye	62 (15)	225 (28)	287 (23)
Liver	68 (16)	182 (22)	250 (20)
Bone Marrow	29 (7)	66 (8)	95 (8)
Spleen	41 (10)	52 (6)	93 (7)
Bone/Joint	30 (7)	53 (7)	83 (7)
Ear, Nose, Throat	33 (8)	87 (11)	120 (10)
Parotid, Salivary gland	13 (3)	21 (3)	34 (3)
Muscle	5 (1)	7 (1)	12 (1)
Hypercalcemia	42 (10)	47 (6)	89 (7)

* Judson MA. Sarcoidosis Vasc Diff Lung Dis 2012; 29:119–127 (Reference [4]).

**Table 2:** A summary of granulomatous conditions other than sarcoidosis.

General Categories	Specific Conditions
**Infections**	
Mycobacteria	Tuberculosis
	Non-tuberculous mycobacteria
Fungi	Cryptococcus
	Histoplasmosis
	Blastomycosis
	Coccidioidomycosis
Other infections	Mycoplasma
	Pneumocystis jiroveci
	Brucellosis
	Toxoplasmosis
	Leishmaniasis
	Schistosomiasis
	Bartonella
	Mononucleosis (Epstein Barr virus)
	Cytomegalovirus
	Coxiella burnetii (Q fever)
	Treponema (syphilis, yaws)
**Environmental and occupational exposures**	
Hypersensitivity pneumonitis	
Pneumoconioses	Beryllium (chronic beryllium disease)
	Titanium
	Aluminum
**Malignancies**	Lymphoma
	Sarcoidosis-like reaction of malignancy
**Vasculitidies/Connective tissue diseases**	Granulomatosis with polyangiitis
	Rheumatoid nodules
**Localized granulomatous reactions to foreign substances**	Lung aspiration
	Foreign body reactions
**Drug-induced sarcoidosis-like reactions (DISRs)**	Highly active retroviral therapy (HAART)
	Immune checkpoint inhibitors
	Tumor necrosis alpha antagonists
	Interferon
**Diffuse granulomatous reactions from an autoimmune inflammatory syndrome induced by adjuvants**	
**Granulomatous lesions of unknown significance (GLUS syndrome)**	
**Granulomatous interstitial lung disease (GLILD) related to common variable immunodeficiency (CVID)**	
**Necrotizing sarcoid granulomatosis**	
**Blau syndrome**	
**Orofacial granulomatosis**	
**Crohn’s disease**	
**Primary biliary cirrhosis**	

**Table 3:** Environmental and occupational risk factors and protective factors for sarcoidosis.

Risk Factor Exposure/Occupation	Location and/or Study Population	Reference
Spring season (disease onset)	Rochester, MN; Turkey; New Zealand; Catalonia, Spain	[100,101,102,103]
Summer season (disease onset)	USA Veterans	[104]
Fall season (disease onset)	Rochester, MN	[105]
Specific regions of Ireland	Ireland	[106]
Northern latitudes	Ireland	[106]
Northern latitudes	Japan	[83]
Southeast United States	United States	[107]
Coastline of South Carolina	South Carolina	[108]
Living in forest or arable land	Poland	[109]
Living near areas with metal industries	Switzerland	[110]
Living in areas with potato production, artificial meadows, grain production	Switzerland	[110]
Firefighters	NYC	[111]
Firefighters	Providence, RI	[112]
Ship servicemen	US Navy	[113]
Aviation structural mechanics	US Navy, AA	[113]
Culinary specialists	US Navy, W	[113]
Using insecticides	USA	[114]
Musty odors at work	USA	[114]
Building materials	USA	[115]
Hardware	USA	[115]
Garden supplies	USA	[115]
Mobile homes	USA	[115]
Industrial organic dusts	USA	[115]
Education	Detroit, AA	[116]
Metal machining	Detroit, AA	[116]
Metalworking	Detroit, AA	[116]
Transportation services	Detroit, AA	[116]
Construction workers	Sweden	[117]
Silica (metal-halide lamp production)	N/A	[118]
Photocopier toner	USA, AA	[119]
World Trade Center dust	FDNY	[120]
Working in high humidity	Detroit MI, AA	[116]
Titanium	Detroit MI, AA	[116]
Vegetable dust	Detroit MI, AA	[116]
Man-made mineral fibers	N/A	[121]
Woodstove use	SC	[122]
Fireplace use	SC	[122]
Musty odor exposure	Detroit MI, AA	[116]
Non-public water use	SC	[122]
Living/working on a farm	SC	[122]

AA: African American; W: white; N/A: not applicable; FDNY: Fire Department of New York City; SC: South Carolina.
